# The role of *Anvillea garcinii* and its compounds in health and disease: An overview

**DOI:** 10.22038/ajp.2024.25131

**Published:** 2025

**Authors:** Ali Zarei, Fatemeh Rasekh, Samaneh Ahmadpour Khorrami, Behnam Masmouei, Saeed Changizi-Ashtiyani, Majid Ramezani, Amirhossein Zarei

**Affiliations:** 1 Department of Physiology, Estahban School of Paramedical Sciences, School of Nursing Hazrat Zahra (P.B.U.H) Abadeh, Shiraz University of Medical Sciences, Shiraz, Iran; 2 Department of Biology, Payam Noor University of Tehran, Tehran, Iran; 3 Department of Chemical Engineering, Shiraz Branch, Islamic Azad University, Shiraz, Iran; 4 Department of Nursing, School of Nursing Hazrat Zahra (P.B.U.H) Abadeh, Shiraz University of Medical Sciences, Shiraz, Iran; 5 Department of Physiology, Iran University of Medical Sciences, Tehran, Iran; 6 Department of Internal Medicine, Baqiyatallah University of Medical Sciences, Tehran, Iran; 7 Department of Education, Abadeh Vesal High School, Abadeh, Iran

**Keywords:** Anvillea garcinia, Health, Anti-oxidant, Diabetes, Cancer

## Abstract

**Objective:**

This scoping review aims to examine the potential health benefits of *Anvillea garcinii* and its compounds and provide recommendations based on available research. *A. garcinii* is a plant species in the daisy family that has demonstrated several therapeutic and preventive effects.

**Materials and Methods:**

This review was conducted with a comprehensive approach. We meticulously searched multiple databases, including PubMed, Embase, Scopus, Cochrane, SID, and Magiran, using the keyword "*A. garcinii* " on October 4, 2023.

**Results:**

Research suggests that *A. garcinii* extract possesses several properties that could benefit health. These include anti-hyperglycemic, anti-hyperlipidemic, and anti-inflammatory activities. The extract also displays anti-oxidant properties, enhances insulin sensitivity, and inhibits α-amylase and α-glucosidase. Additionally, it exhibits hepatoprotective activity, cytotoxic activity against cancerous cells, anti-fungal, anti-human immunodeficiency virus (HIV), anti-bacterial, anti-cholinesterase, and anti-tyrosinase activities.

**Conclusion:**

The diverse health benefits of *A. garcinii* extract and its active compounds, such as germacranolide and parthenolide, present significant potential for use in the food, cosmetic, and pharmaceutical industries. This potential, especially in treating diabetes, gastric ulcers, and cancer, opens up exciting possibilities for the future.

## Introduction

The use of medicinal plants in treating diseases has a rich historical background, connecting us to our traditional roots. *Anvilleais* is a genus of flowering plants in the daisy family, including *A. garcinii,* which is a shrub with yellow flowers and includes four species distributed in North Africa to Iran in several countries in the Middle East such as Egypt, Palestine, and Saudi Arabia local people widely use* A. garcinii* for its medicinal properties. It is traditionally used to treat hemorrhagic diarrhea, gastrointestinal disorders, hepatitis, pulmonary diseases, the common cold, and hepatic disease (Hammiche and Maiza, 2006; Miara et al., 2019). 

Studies have found that *A. garcinii* 's aerial parts contain various beneficial compounds, including phenolic compounds, flavonoids, and germacranolides. Notably, 9-hydroxy parthenolide is among these compounds and is a reliable source in the pharmaceutical and cosmetic industries. Additionally, it has been used in the synthesis of various chemical compounds. (Branch, 2015; Perveen et al., 2018; Abdel-Sattar and McPhail, 2000; Moumou et al., 2011a-c, 2010, 2012, 2011d). 

The extract of the aerial parts of *A. garcinii *shows anti-oxidant, insulin sensitivity increasing, and α-amylase and α-glucosidase inhibitory properties, as well as anti-hyperglycemic, anti-hyperlipidemic, and anti-inflammatory activities. It is helpful in the treatment of diabetes and its complications (Kandouli et al., 2017; Moumou et al., 2012; Moumou et al., 2011d). 

An ethanol extract of *A. garcinii *has been shown to have hepatoprotective effects against CCl4-induced hepatotoxicity in rats (Perveen et al., 2018; Zarei et al., 2013).

 It also has a powerful anti-ulcer effect (Perveen et al., 2018; de Lira Mota et al., 2009). Ethylacetate extract of this plant has anti-oxidant, anti-cholinesterase, anti-tyrosinase, and anti-α-glucosidase (Saoud et al., 2019) activities and the extracted polysaccharide of *A. garcinii *exerts a substantial anti-oxidant effect on neutrophils (Boukemara et al., 2016). Chloroform and n-butanol fractions of *A. garcinii* show significant cytotoxic activity in colon cancer (HCT-116), hepatocellular carcinoma (HePG-2), HeLa cervix cells (HeLa), and lung cancer (A-549). In addition, epoxyparthenolide and 9ahydroxyparthenolide show significant cytotoxic effects on different cancerous cell lines (Perveen et al., 2018; Zarei et al., 2013). Essence and extract of the aerial parts of *A. garcinii *have anti-HIV (Kolev et al., 2014), anti-fungal, and anti-bacterial properties (Perveen et al., 2019) and prevent form metal corrosion in acidic environments (Al-Otaibi et al., 2014). *A. garcinii* powder may permanently replace chemical pesticides due to its inhibitory effect on the growth of *Penicillium italicum* in Citrus (Askarne et al., 2012). Consequently, many preventive and therapeutic effects of *A. garcinii *have been observed, though these effects have not been systematically reviewed. The current study reviews the role of *A. garcinii *and its compounds in health and disease.

## Materials and Methods

This review was conducted by searching the *A. garcinii *keyword in valid scientific databases. This keyword was searched in PubMed (11 records), Embase (9 records), Scopus (45 records), and Cochrane (0 records). In addition, SID (8 records) and Magiran (4 records) were searched to access Iranian articles. An article search was performed on 4 October 2023. Other methods found six themes, such as reviewing references in the articles. Collectively, 77 articles were found. After removing duplicates (22 records) and irrelevant articles (17 records), 38 articles were evaluated in this study.

## Results

### Composition of A. garcinii

The essence of the vegetative parts and flowers of *A. garcinii *consists of 140 compounds. One hundred twenty-six compounds in flowers and 119 in the vegetative parts account for 95.7% and 94.9% of the whole compounds, respectively. The main compounds in the flower essence are bornyl acetate (33.7%), cis-nerolidol (7.3%), and camphene (6.1%). The major compounds of the green or vegetative parts of *A. garcinii *include cis-nerolidol (16.0%), terpinene-4-ol (10.4%), and cabreuva oxide B (6.4%) (Khan et al., 2016; Rustaiyan et al., 2011). Results of another study suggest that 97.44% of the whole compounds of the essence of the leaves, flower, and other parts of *A. garcinii *are composed of only 25 compounds, which most notably include myristicin (58.79%), Bicyclo (5.3.0) decane, 2-methylene-5-(1-methyl vinyl)-8-methyl (7.71%), 5-Methyl-4-nonene (3.46% (E)-Ocimene (3.39%) (Oucheikh et al., 2022). 

Additionally, *A. garcinii* boasts palmitic acid, stigmast-5-en-3-vol, and cholestan-3-one-4,4-dimethyl, as noted by Al-Otaibi et al. in 2014. Furthermore, its aerial parts are a dependable source of 9-hydroxy parthenolide, found in the pharmaceutical and cosmetic sectors, as reported by Moumou et al. in various studies.

Anvillea essence contains a significant amount of phenolic compounds (Branch, 2015) and its leaves includes flavonoids such as hispidulin, nepetin, jaceosidin, spinacetin, spinacetin 7-glucoside, patuletin 7-glucoside, spinacetin 3-glucoside, kaempferol 6-methyl ether 3- glucoside, quercetin 3-glucoside, patuletin 3-diglucoside, isorhamnetin 3-diglucoside, quercetin 3-rhamnoglucoside, quercetin 3-diglucoside 7glucoside, isorhamnetin 3-rhamnoglucoside, quercetin 3-glucoside3,4,dimethyl ether, 6-methoxy kaempferol 3-galacoside, 6-methoxy kaempferol 3-galactoside 7-methyl ether, 6-methoxy kaempferol 3-galactoside 7,4dimethyl ether, 6-methoxy kaempferol 3-rhamnoglucoside, 6-methoxy quercetin 3- rhamnoglucoside 3 methyl ether, 6-methoxyapigenin, and 6-methoxylutolin (Ulubelen et al., 1979; Dendougui et al., 2006), nepetin, isorhamnetin and jaceosidin and chlorophyll pigments (Destandau et al., 2015) 9α-hydroxyparthenolide-9-O-β-D-glucopyranoside, spinacetin 3-O-[α-L-rhamnopyranosyl-(1→6)-βD-glucopyranoside]-7-O-[α-L-rhamnopyranoside], kaempferol 3-Orutinoside, kaempferol 7-O-β-D-glucopyranoside, quercetin7-O-β-D-glucopyranoside (Perveen et al., 2018).

The aerial parts of *A. garcinii *contain germacranolides, including 9β -hydroxy parthenolide and 9α-hydroxy parthenolide (Destandau et al., 2015; Tyson et al., 1981) and cis-parthenolid-9-one (Abdel-Sattar et al., 2000) 9α-hydroxy parthenolide is one of the most important compounds of *A. garcinii *from which several sesquiterpene compounds such as 9α-Acetoxy-1β, 10α-epoxy parthenolide (with molecular formula C17H22O6) have been synthesized (Moumou et al., 2010; Moumou et al., 2012; Moumou et al., 2011d).

Authors have reported four new compounds named garcinamine. Garcinamine B is similar to garcinamine A. The only difference is that in C-13, the L-phenylalanine amino acid in garcinamine A is replaced with the L-valine amino acid in garcinamine B. Also, garcinamine C is similar to garcinamine A and B, with the only difference of C-13 amino acid (proline). Garcinamine D is identical to garcinamine C, and the position of OH bounded to C-9 has been changed (Table 1).

### Anti-diabetic and anti-hyperlipidemic properties

Hyperlipidemia may lead to a series of diseases by causing vital organ dysfunction, and medicinal plants containing various active compounds help to treat hyperlipidemia (Changizi-Ashtiyani et al., 2017; Changizi-Ashtiyani et al., 2018; Changizi-Ashtiyani et al., 2013). The aerial parts of *Anvillea garcinia *contain two hypoglycemic compounds, 9α-hydroxy parthenolide and 9β –hydroxy parthenolide (Abdel Sattar et al., 1996; Ulubelen et al., 1979). By studying the effects of *A. garcinii *plant extract (300 mg/kg for 40 days) in diabetic rats, it was found that this plant showed significant anti-diabetic potential by reducing the amount of glucose and maintaining body weight and concentration of lipid profiles (Kharjul et al., 2014). The administration of the extract of other species of this plant, including Anvillea (150 mg/kg for 12 weeks), also led to body weight control and improved insulin and glucose levels. Because, on the one hand, they have anti-oxidant properties; on the other hand, they inhibit alpha-amylase and alpha-glucosidase enzymes (Kandouli et al., 2017) (Table 2).

### Effect on thyroid activity

The experimental investigation of the effect of* A. garcinii *extract on thyroid hormone levels in rats showed that consumption of *A. garcinii *plant extract (100 and 300 mg/kg) led to an increase in thyroid hormones and a decrease in TSH, cholesterol, and other lipid profiles. Usually, the relationship between lipid profiles and thyroid hormones is inverse. This plant extract can also reduce blood fat by increasing thyroid hormones. The hypolipidemic effects of this plant may also be due to the presence of phenolic and flavonoid compounds. In addition, this plant extract prevents the decrease of thyroid hormones by reducing tyrosinase enzyme activity and dopamine (Rasekh et al., 2022).

### Hepatoprotective activity

A study evaluated the hepatoprotective effects of ethanol extract, n-butanol, and chloroform fractions of *A. garcinii* leaves and silymarin in rats with CCl4-induced liver damage. The rats were given different dosages of the extracts and silymarin before being intoxicated with CCl4. The study revealed that two days of pre-treatment with silymarin (10 mg/kg) and ethanol extract of *A. garcinii* significantly reduced the levels of aspartate aminotransferase (AST), alanine aminotransferase (ALT), gamma-glutamyl trans-peptidase (GGT), alkaline phosphatase (ALP), malonaldehyde (MDA), and bilirubin in the rats' serum and increased serum protein levels. However, the n-butanol fraction showed no protective effect in both dosages. On the other hand, a higher dose of chloroform fraction showed better improvement in all serum parameters compared to silymarin.

The hepatic histology of rats treated with total ethanol extract of *A. garcinii *with a 400 mg/kg dose showed some protection in multiple areas of necrosis, hepatic cell washout, and fatty changes. The best result was obtained in the rats treated with 400 mg/kg chloroform fraction, with minimal necrosis and histologic changes. No significant histologic changes were observed in other cases, which may be due to the difference in the composition of the extracts (Perveen et al., 2018). The hepatoprotective effects of these extracts are potentially caused by hepatoprotective compounds such as sesquiterpene and anti-oxidant compounds (Zarei et al., 2015). Likely, a high-fat diet increases insulin levels in diabetic animals. With diabetes treatment and increased insulin sensitivity, the hepatic glycogen content increases, and the insulin level declines to reach its average level (Perveen et al., 2018) (Table 2). 

### Cytotoxic and anti-tumoral activity

A study was conducted on eight compounds of *A. garcinii*, which included garcinamine A (1), garcinamine B (2), garcinamine C (3), garcinamine D (4), parthenolide-9one (5), 9a-hydroxy-1b, ten a-epoxy parthenolide (6), 9ahydroxyparthenolide (7), nine b-hydroxy parthenolide (8), ethanol extract, chloroform fraction, and n-butanol fraction. The study aimed to assess their cytotoxic effects on six different cancer cell lines, which included breast adenocarcinoma (MCF-7), colon carcinoma (HCT-116), hepatic carcinoma (HePG-2), HeLa cervix cells (HeLa), and lung cancer (A-549), and to compare them with vinblastine. 

The study showed that the ethanol extract had low cytotoxic effects, whereas the chloroform and n-butanol fractions showed significant cytotoxic effects against colon, liver, cervix, and lung cancer. The chloroform fraction was found to be highly effective compared to vinblastine. Compounds 6 and 7 showed significant cytotoxic effects against different cancer cell lines, and the cytotoxic effects of compound 6 were even more prominent compared to vinblastine in the cervix and hepatic cancer. 

Furthermore, after comparing the combinations of compounds 1 (containing L-phenyl alanine), 2 (containing L-valine), and 3 (containing L-proline), it was found that compound 3 had the highest cytotoxic effects due to the presence of L-proline amino acid (Kolev et al., 2014). It was observed that sesquiterpene lactone bonds with different amino acids, which affects its cytotoxic effects. 

In conclusion, parthenolide has attracted particular attention as an anti-cancer agent due to its promising potential (Perveen et al., 2018). 

Germacranolides driven from the aerial parts of *A. garcinii *show anti-tumoral effects (Abdel Sattar et al., 1996). Three derivatives of guaiane sesquiterpenes (garcinamines C and D and nine β-hydroxy parthenolides) were obtained in the chromatography of *A. garcinii *leaves. These compounds show significant effects against lung cancer, colon carcinoma, and breast cancer (Aati et al., 2021).

### Anti-ulcer effects

The ethanol extract of *A. garcinii *leaves shows potent anti-ulcer effects, providing the highest protection for gastrointestinal mucosa against ulcerating agents. Different mechanisms of action may be involved in this protective effect, including a cytoprotective and anti-secretory mechanism that improves mucosal blood flow. However, the anti-oxidant effects of the compounds of ethanol extract of *A. garcinii, *including free radical modification, inhibition of oxidizing enzymes, and reduced lipids peroxidation, are involved in the underlying mechanism of the prominent anti-ulcer effects. Histologic and laboratory results of studies proved that it reduced MDA and increased protein levels in ethanol-induced gastric ulcers (de Lira Mota et al., 2009). These findings indicate the preventive effects of *A. garcinii *ethanol extract (400mg/kg) against ulcerative colitis in Wistar rats. These effects may be attributed to the anti-inflammatory and anti-oxidant effects of the active biochemical of the section (Perveen et al., 2018) (Table 3).

### Anti-microbial properties

The aerial parts of *A. garcinii *includes 9α-hydroxyparthenolide, 9β-hydroxyparthenolide, 9β-hydroxy 1 β,10α epoxyparthenolide, 9α-hydroxy-1 β,10 α epoxyparthenolide and parthenolid-9-one. These compounds show *in vitro* anti-HIV effects (Abdel Sattar et al., 1996). *A. garcinii *leaves show anti-microbial effects due to guaianolide sesquiterpenoids, six of which have been identified. They all show anti-bacterial effects against Gram-positive and Gram-negative bacteria (*Staphylococcus aureus* and *Escherichia fergusonii*) and anti-fungal effects against *Candida albicans* and Candida* parapsilosis* fungi. (Perveen et al., 2019; Perveen et al., 2020).

Researchers evaluated the anti-bacterial effects of methanol and ethyl acetate extract of *A. garcinii *against pathogen species such as *Staphylococcus aureus*, *Bacillus subtilis*, *Salmonella abony*, and *Escherichia coli*. They reported promising anti-bacterial effects, especially for the methanol extract. Thus, these compounds may be an additive in the food, cosmetic, and pharmaceutical industries (Rustaiyan et al., 2011; Mohamed et al., 2015; Mahdjar et al., 2019). *A. garcinii *essence, containing phenols and monoterpenes, shows inhibitory effects on Gram-negative bacteria against *Staphylococcus* *epidermidis* and *Staphylococcus saprophyticus*. *Staphylococcus epidermidis* and *Staphylococcus saprophyticus* (through changing membrane permeability and osmolarity imbalance) and anti-fungal effects against fungi such as *Klebsiella oxytoca* and *Fusarium solani* (Rustaiyan et al., 2011), (Mehdi, AYM. 2011). *A. garcinia *10% powder may permanently replace chemical pesticides due to its inhibitory effect on the growth of *Penicillium italicum* in the *Citrus* (Askarne et al., 2012) (Table 3).

### Other properties

Ethyl acetate extract of *A. garcinii *has anti-oxidant and robust free radical scavenging effects. Two germacranolids compounds derived from *A. garcinii*, 9αhydroxyparthenolide 9β- and 9αhydroxyparthenolide, and ethyl acetate extract of *A. garcinii *exert anti-cholinesterase and anti-tyrosinase effects. Inhibition of the anti-tyrosinase reduces melanin synthesis, which may be helpful in skin whitening. 3,5-o-caffeoylquinic acid, derived from *A. garcinii*, shows substantial inhibitory effects on the α-glucosidase development. The first two compounds show significant cytotoxic effects against MCF-7 cancer cell lines. Thus, this plant may be a rich anti-oxidant source in the food industry (Saoud et al., 2019). The free radical scavenging activity of *A. garcinii *is attributed to polyphenolic compounds such as myristicin (Mehdi, AYM. 2011). The extracted polysaccharide of *A. garcinii *exerts a solid anti-oxidant effect on neutrophils. Although neutrophils play a role in identifying, engulfing, and destroying pathogens by oxidative and non-oxidative mechanisms, their high activity leads to tissue damage and inflammatory reactions. Therefore, inhibiting neutrophil activity is an exciting strategy for developing new anti-inflammatory agents. Polysaccharides isolated from *A. garcinii *plants inhibit N-formyl-methionyl-leucylphenylalanine (fMLF) and phorbol myristate acetate (PMA)-induced superoxide anion (O2.) production in human neutrophils as well as PMA-induced Protein kinase C activation. In addition, this plant extract prevents the degranulation of myeloperoxidase (MPO). These results indicate that the polysaccharides isolated from *A. garcinii *can have a strong anti-inflammatory effect by inhibiting the function of neutrophils and by limiting the release of reactive oxygen species (ROS) to the adjacent tissues (Boukemara et al., 2016) (Table 3). 

## Discussion

*A. garcinii* is a plant that contains compounds like hydroxy parthenolide and 9-β-hydroxy parthenolide, which help treat induced and blood disorders. These compounds help to regulate alpha-amylase, alpha-glucosidase, and insulin levels, making the plant effective for treating diabetes. The plant extract is also believed to increase thyroid growth, possibly explaining its hypolipidemic properties. 

*A. garcinii* is also rich in sesquiterpenes, which serve as anti-oxidants and make the plant extract hepatoprotective. The extract also contains anti-fungal, anti-viral, and anti-bacterial properties, making it suitable for use in the food and cosmetic industries. The plant extract is known to reduce the synthesis of melanin, which brightens the skin, and it has anti-tumor and cytotoxic properties due to its germacranolides and terpenes content.

Despite the numerous studies conducted on *A. garcinii*, there is still much to learn about its effects on catecholamine levels, melanin, wound healing, the pituitary-thyroid axis, fatty liver, spermatogenesis, oogenesis, and other areas. 

**Table 1:** Composition of A. garcinii

Composition of flowers			Composition of essential oil for Aerial parts (in south-east of Morocco) Yazdi, 2011	Composition of leaves (in the Persian Gulf of Iran) Rustaiyan et al., 2011)
**1, 3, 5, ** **7-Cyclooctatetraene**	*endo*-Fenchyl acetate	Phytol	6-Methyl-5-hepten-2-one	*α-Pinene*
**(3** ***Z*** **)-4,8-Dimethyl-3,7-nonadien** ***-*** **2-one**	*epi*-*α*-Cadinol	Pinocarvone	β-Pinene	Sabinene
**(4** ***R*** **, 8** ***S*** **)-** ***p*** **-Menth-1-en** ***-*** **9-ol**	Eudesma-4(15),7-dien*-*1-*β*-ol	*p*-Mentha-1,4,8-triene	p-Cymene	*α-Phellandrene*
**(Z, E)-Farnesol**	*exo-Arbozolc*	Sabinene	1,8-Cineole	*p-Cymene*
**1,8-Cineole**	Geraniol	*Sesquiterpene hydrocarbons*	Linalool	Limonene
**1-Tetradecanol**	Geranyl tiglate	Sesquithuriferol	Camphre	1,8-Cineole
**2-Methyl butyl benzoate**	Helifolen*-*12-al Bc	Terpinen-4-ol	trans-Pinocarveol	*α-Terpineol*
**4,8-Dimethyl-nona-3,8-dien** ***-*** **2-one**	Himachalol	Tetradecanal	Isoneral	Bornyl acetate
**7-** ***epi*** **-** ***α*** **-Selinene**	Intermedeol	Tetradecano0.6ic acid	Borneol	7-*epi*-*α*-Selinene
**8,8-Dimethyl-9-methylene-1,5-cycloundecadiene**	*iso*-Ascaridolec	Thujyl acetate	Terpinen-4-ol	α-terpinene
***Aliphatic hydrocarbons***	*iso*-Borneol	*trans-*Calamenene	Myrtenol	β-phellandrene
***Aromatics***	*iso*-Butyl benzoate	*trans-*Carveol	trans-Carveol	o-mentha-1,4,8-trien
**Borneol**	*iso*-Dihydrocarveol	*trans-*Carvyl acetate	Carvone	p-cymene-2-ol
**Bornyl acetate**	Kessane	*trans-*Caryophyllene	Geraniol	α-cadinene
**Cabreuva oxide A, B,C and D**	Khusimone	*trans-*Nerolidol	cis-Chrysanthenyl acetate	α-curcumene
**Camphene**	Lavandulol	*trans-*Pinocarveol	Acide nonanoic	*γ*-curcumene
**Camphene hydrate**	Lavandulyl-2-methyl butanoate	*trans-p*-Mentha-2,8-dien*-*1-ol	Bornyl acetate	Germacrene-A
**Camphor**	Liguloxide	*trans-*Tagetenone (*E*-Ocimenone)	Carvacrol	(trans)-b-elemenone
**Caryophylla-4-(14),8(15)-Dien** ***-*** **5-** ***α*** **-ol**	Limonene	*Z-α-Santalol*	Myrtenyl acetate	humulene epoxide
**Caryophyllene oxide**	Linalool	*α-Copaene*	Piperetenone oxyde	-
**Chrysanthenone epoxide**	Methyl tetradecanoate	*α-Eudesmol*	α-Humulene	-
***cis*** **-1(7),8-** ***p*** **-Menthadien** ***-*** **2-ol**	*Monoterpene hydrocarbons*	*α-Funebrenec*	γ-Muurolene	-
***cis*** **-13-Octadecen** ***-*** **1-yl acetate**	Myrtenol	*α-Muurolol*	isobutyrate de geranyle	-
***cis*** **-3-Hexenyl benzoate**	Myrtenyl acetate	*α-Patchoulene*	γ-Cadinene	-
***cis*** **-Carvyl acetate**	*n-Decanal*	*α and β -Pinene*	Kessane	-
***cis*** **-Dracunculifoliol**	Nerol oxide	*α-Selinene*	6-oxo cyclo nerolidol	-
***cis*** **-Jasmone**	*n-Heptadecane*	*α-Sinensal*	cis 8-acetoxychrysantenyl acetate	-
***cis*** **-Methyl jasmonate**	*n-Hexyl benzoate*	*α-Terpineol*	Caryophyllene oxyde	-
***cis*** **-Nerolidol**	Nonanal	*α*-Terpinyl acetate	Isovalerate de geranyle	-
***cis*** **-Pinocarvyl acetate**	*Oxygenated aliphatic hydrocarbons*	*β-Bisabolol*	6-hydroxcyclonerolidol	-
***cis*** **-** ***p*** **-Ment-2-en** ***-*** **1-ol**	*Oxygenated monoterpenes*	*β-Dihydroagarofuran*	τ-Cadinol	-
***cis*** **-Verbenyl acetate**	*Oxygenated sesquiterpenes*	*β-Eudesmol*	β-Eudesmol	-
**Cuminaldehyde**	Palmitic acid	*β-Maaliene*	α-Cadinol	-
**Dehydro-1,8-cineole**	*p-Cymene*	*β-Selinene*	Cedroxyde	-
**Dehydro-** ***ar*** **-ionene**	Pentadecanal	*γ-Curcumen-15-al*	α-Oxobisabolene	-
***Diterpenes***	Perilla alcohol	*γ-Eudesmol*	-	-
**Dodecanoic acid**	Perilla aldehyde	*δ-Cadinene*	-	-

**Table 2:** Anti-diabetic and anti-hyperlipidemic properties, effect on thyroid activity and hepatoprotective activity of A. garcinii

**Ref**	**Outcomes**	**Dose**	**Model **	**Extract and constituents**	**Effects**
Perveen et al., 2018	Decrease of ALT and MDA.	200 mg/kg For six days	induced Cytotoxicity in rats by CCl_4_	Total ethanol extract of leaf	Hepatoprotective activity
	Decrease AST, ALT, ALP, GGT, Bilirubin, MDA, Total protein, and Total protein.	400 mg/kg For six days			
	Decrease of AST, ALT, ALP, GGT, Bilirubin and MDA. Increase of NP–SH and Total protein.	200 mg/kg For six days	induced ytotoxicity in rats by CCL_4_	Chloroform fraction of leaf	
	Decrease of AST, ALT, ALP, GGT, Bilirubin and MDA. Increase of NP–SH and Total protein.	400 mg/kg For six days For six days			
	Decrease of ALT, ALP, and Bilirubin.	200 mg/kg For six days	induced Cytotoxicity in rats by CCl_4_	n-Butanol fraction of leaf	
	Decrease of ALT, MDA. Increase of Total protein.	400 mg/kg For six days			
Rasekh et al., 2022	Decrease of Cholesterol, TG, VLDL, and TSH. Increase of T3 and T4	100 mg/kg For 45 days	hypercholesterolemic rats	alcoholic extract of aerial parts	anti-hyperlipidemic and Hyperthyroid activity
	Decrease of Cholesterol, TG, VLDL, LDL, HDL, and TSH. Increase of Increase of T3 and T4.	300 mg/kg For 45 days			
Kharjul et al., 2014	decreasing blood glucose levels and maintaining body weight and serum lipid concentrations to normal	300 mg/Kg For 45 days	streptozotocin-induced diabetic rats	ethanolic extract	Anti-diabetic activity

AST: aspartate aminotransferase, ALT: alanine aminotransferase, ALP: alkaline phosphatase, GGT: gamma-glutamyl trans-peptidase, MDA: malonaldehyde, NP–SH: non-protein sulfhydryl groups, CCL4: carbon tetrachloride, TG; triglyceride, TSH: stimulating thyroid hormone, VLDL: very low-density lipoprotein, LDL: low-density lipoprotein, HDL: high-density lipoprotein, T3: triiodothyronine, and T4: thyroxine

**Table 3:** Cytotoxic and anti-tumoral, anti-ulcer, and other effects of A. garcinii

**Ref**	**Outcomes**	**Dose**	**Model **	**Extract and constituents**	**Effects**
Aati et al., 2021	Garcinamines C and D and 9β-hydroxyparthenolide exerted cytotoxic effects in a dose- and time-dependent manner.	Treatment of cancer cells with different concentrations for 24 hours	human cancer cell lines (A549 lung carcinoma, MCF-7 breast carcinoma, and LoVo colon carcinoma)	guaianolide-proline (garcinamines F–H) isolated from the plant	Anti-cancer activity
Boukemara et al., 2016	Inhibition of neutrophil stimulators (fMLFand PMA) Decreased active ty of NADPH oxidase (NOX2) inhibition of peroxide anion production or o_2_ Inhibiting the production and transfer of PKCβ and p47phox to the cell membrane Inhibition of myeloperoxidase (MPO), CD11b membrane expression and degranulation of neutrophils	different concentration (For example, 300 μg) for 8 min	Neutrophils stimulated by N-formyl-methionyl-leucyl-phenylalanine (fMLF)- and phorbol myristate acetate (PMA)	Polysaccharides isolated from plants	Anti-inflammatory effects
Perveen et al., 2018	decrease in gastric secretion and titratable acidity, in gastric lesion index in pylorus lessened the ulcer index induced by ethanol	400mg/kg for 6 hours	Stomach ulcer caused by ethanol and indomethacin	chloroform and n-butanol fractions	Anti-ulcer activity
Perveen et al., 2019	Inhibition of Candida albicans growth	0.32 μg/ml	* in vitro*	Sesquiterpenes	Anti-fungal activity
	Inhibition of Candida parapsilosis growth	1.4 μg/ml			
	Inhibition of S. aureus growth	1.7 μg/ml	* in vitro*	Sesquiterpenes	Anti-bacterial activity
	Inhibition of E. fergusonii growth	3.5 μg/ml			
Rustaiyan et al., 2011	Inhibition of Staphylococcus Aureus growth	0.4 mg/ml	*in vitro*	Leaf oil	
	Inhibition of Staphylococcus epidermidis growth	0.8 mg/ml			
	Staphylococcus Saprophyticus growth	4.0 mg/ml			
	Inhibition of Shigella flexneri growth	1.8 mg/ml			
	Inhibition of Escherichia coli growth	0.5 mg/ml			
	Inhibition of Staphylococcus Aureus growth	0.6 mg/ml	*in vitro*	flower oil	
	Inhibition of Staphylococcus epidermidis growth	1.2 mg/ml			
	Inhibition of Staphylococcus Saprophyticus growth	5.5 mg/ml			
	Inhibition of Shigella flexneri growth	2.0 mg/ml			
	Inhibition of Escherichia coli growth	0.7 mg/ml			
